# Infantile Hypertrophic Pyloric Stenosis Without Metabolic Alkalosis: A Report of Two Cases

**DOI:** 10.7759/cureus.68548

**Published:** 2024-09-03

**Authors:** Ruhi Shaligram, Sudhir Malwade, Balakrushna P Garud, Shailaja Mane

**Affiliations:** 1 Pediatrics, Dr. D.Y. Patil Medical College, Hospital and Research Centre, Pune, IND

**Keywords:** metabolic acidosis, gastric outlet, pylorus, hypertrophy, infantile hypertrophic pyloric stenosis

## Abstract

Infantile hypertrophic pyloric stenosis (IHPS) is a condition typically characterized by hypertrophy of the pylorus, leading to gastric outlet obstruction and forceful, nonbilious vomiting in young infants. This case series reports two infants with IHPS who exhibited metabolic acidosis, deviating from the classical biochemical presentation of hypochloremic, hypokalemic metabolic alkalosis. The unusual occurrence of metabolic acidosis in these cases suggests the possibility of alternative or additional pathophysiological mechanisms at play. Such deviations from the expected biochemical profile highlight the complexity of IHPS and the need for a broader diagnostic perspective.

## Introduction

Infantile hypertrophic pyloric stenosis (IHPS) is a condition that affects young infants and is characterized by the thickening of the pylorus, which can nearly obstruct the gastric outlet, leading to forceful vomiting [1]. Typically, IHPS presents in infants aged between two to six weeks with immediate postprandial vomiting. The vomiting is nonbilious, forceful, and often described as "projectile," accompanied by weight loss [2]. A significant decrease in the activity of neuronal nitric oxide synthase (nNOS) in the pyloric muscle is observed in IHPS. This reduction in nitric oxide (NO) production leads to impaired relaxation of the pyloric sphincter, contributing to hypertrophy and hyperplasia of the pyloric muscle [3]. Consequently, the pyloric sphincter remains contracted, causing functional gastric outlet obstruction. This lack of relaxation increases gastric pressure and peristaltic activity, culminating in the characteristic projectile vomiting seen in affected infants [1,3].

The classic electrolyte disturbance in pyloric stenosis is hypochloremic, hypokalemic metabolic alkalosis attributed to the loss of gastric hydrochloric acid [1]. Infants with prolonged IHPS symptoms typically exhibit low serum chloride and potassium levels, along with elevated bicarbonate levels, a condition referred to as hypochloremic alkalosis. Clinically, an "olive-like" mass may be palpated in the right upper quadrant of the abdomen [4].

Globally, IHPS has a reported incidence of approximately one to three per 1,000 live births, with prevalence varying by geographical region and ethnicity. It is more common among Caucasians than African and Asian populations [5]. The condition occurs more frequently in males than females, with a male-to-female ratio ranging from 3:1 to 5:1. Some studies have noted a decline in the incidence of IHPS in recent decades [6].

In India, the incidence of IHPS is estimated to be lower than in Western countries, approximately 0.4 to 0.7 per 1,000 live births [7]. Consistent with global trends, the condition is more common in males. Although precise prevalence data in India is limited, available studies suggest a male predominance and a lower overall incidence than in Western countries.

A family history of IHPS significantly increases the risk, indicating a strong genetic predisposition, with higher rates observed in first-degree relatives of affected individuals. Seasonal variations and exposure to certain environmental factors have been suggested, although these associations are not well-established.

## Case presentation

Case 1

We present the case of a late preterm male neonate, born at 35 weeks gestation with a birth weight of 1.9 kg. He was the first of a dichorionic diamniotic (DCDA) twin pregnancy, delivered via normal vaginal delivery. His twin, also a male, was born with a birth weight of 1.7 kg. On the 25th day of life, corresponding to a corrected gestational age of 38.4 weeks, twin 1 exhibited several concerning symptoms. The infant presented with lethargy, multiple episodes of projectile and non-bilious vomiting, and difficulty accepting feeds. Additionally, he had not been passing stools and was noted to have Grade 2 dehydration. In response to these symptoms, we initiated intravenous (IV) fluids and provided supportive management to stabilize the neonate, addressing his dehydration and feeding difficulties. A blood gas investigation (Table 1) was done and analysis revealed mild metabolic acidosis.

**Table 1:** Blood investigation (Case 1)

Parameter	Value	Normal range
pH	7.27	7.35 - 7.45
pCO2	44 mmHg	35 - 45 mmHg
pO2	95 mmHg	80 - 100 mmHg
Lactate	0.3 mmol/L	0.5 - 2.2 mmol/L
HCO3-	20.2 mmol/L	22 - 26 mmol/L
Base excess	-6.7 mmol/L	-2 to +2 mmol/L
Sodium	137 mmol/L	135 - 145 mmol/L
Potassium	4.96 mmol/L	3.5 - 5.0 mmol/L
Chloride	113 mmol/L	98 - 106 mmol/L

**Figure 1:**
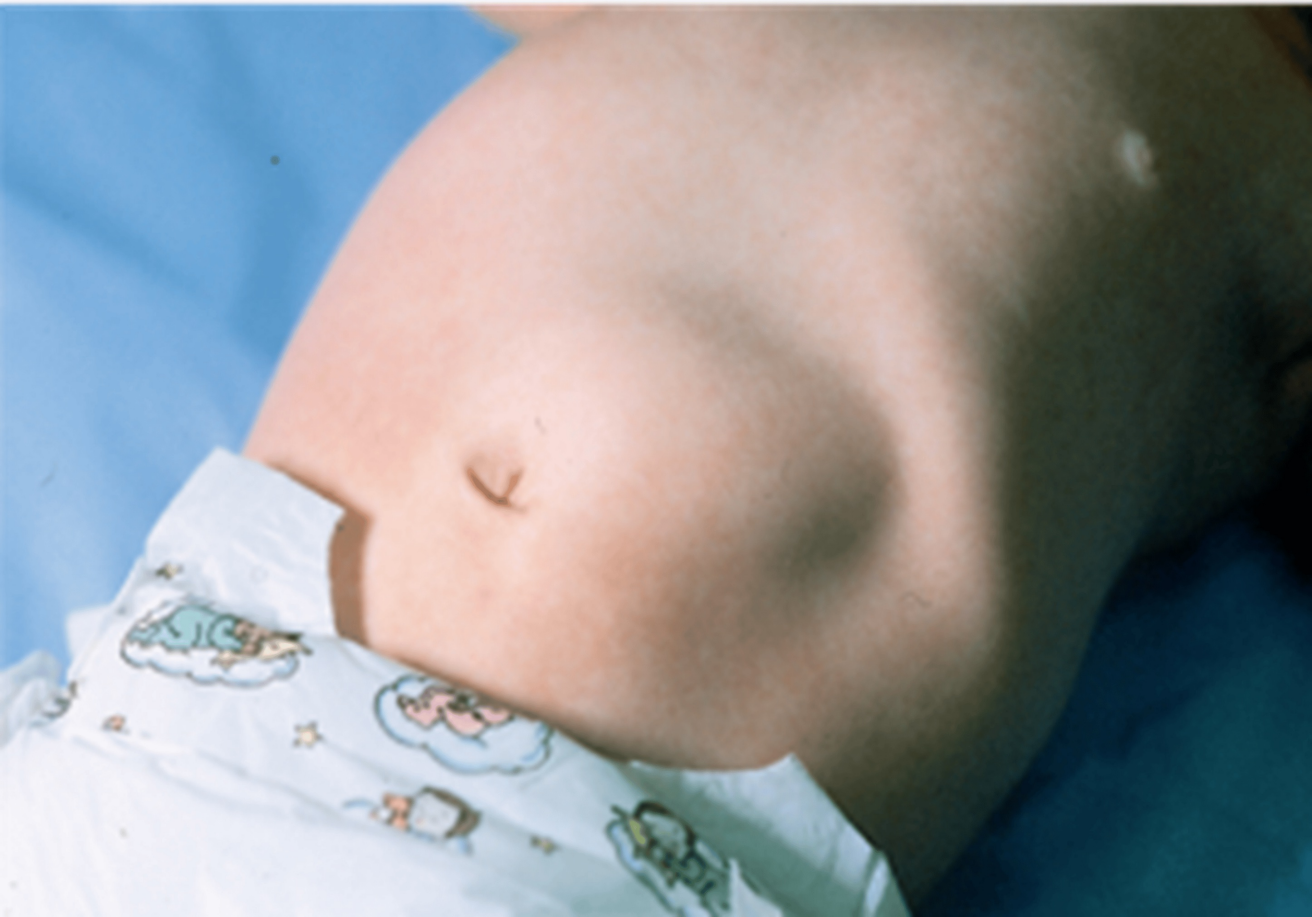
Clinical picture of the patient (Case 1) The classically described "olive-like" mass in the right upper quadrant.

**Figure 2:**
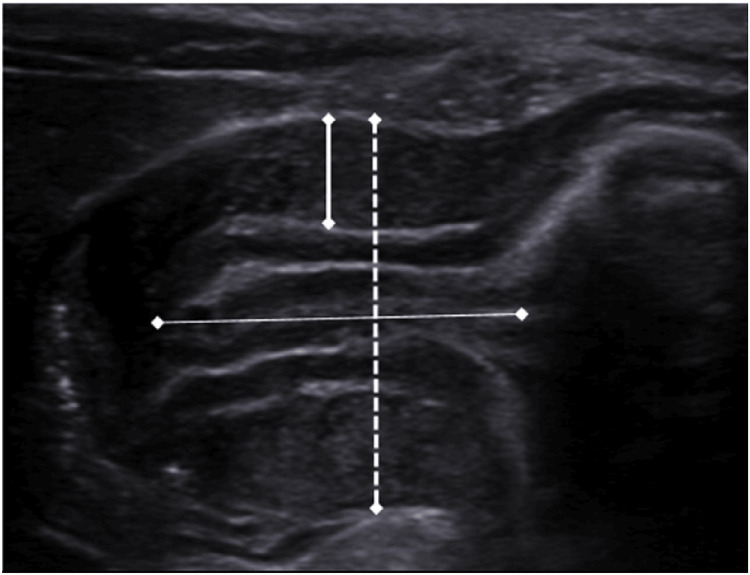
USG abdomen (Case 1) Thickened pylorus measuring 4.5 mm and a symmetrically thickened and edematous wall of length 15 mm, with luminal narrowing. USG, ultrasound

The "olive-like" mass in the right upper quadrant was appreciated (Figure 1). However, the initial ultrasound (USG) of the abdomen and pelvis showed only gaseous shadows, which was misleading given the clinical symptoms suggestive of IHPS. Despite the inconclusive arterial blood gas (ABG) and USG findings, clinical judgment led to a repeat abdominal USG three days later. The repeat USG showed a thickened pylorus measuring 4.5 mm and a symmetrically thickened and edematous wall of length 15 mm, with luminal narrowing and absent peristalsis, consistent with IHPS (Figure 2). The patient subsequently underwent a Ramstedt pyloromyotomy, which was uneventful. Ramstedt pyloromyotomy involves cutting through the outer layer of the pyloric muscle sparing the inner muscular layer, which allows the underlying mucosa to bulge outward, widening the pyloric canal and relieving the obstruction. Later, the patient was started on feeds 72 hours post-surgery, with successful recovery.

Case 2

A female neonate, born at 38 weeks gestation via normal vaginal delivery with a birth weight of 2 kg, presented at 22 days of age with a clinical picture suggesting late-onset sepsis. The neonate exhibited symptoms of lethargy, refusal of feeds, multiple episodes of projectile vomiting with a brown coloration, brown-colored RT aspirate, abdominal distension, and Grade 2 dehydration. The ABG (Table 2) analysis revealed a metabolic acidosis.

**Table 2:** Blood investigation (Case 2) BE, base excess

Parameter	ABG result	Normal range
pH	7.34	7.35-7.45
pCO2	31 mmHg	35-45 mmHg
pO2	40 mmHg	75-100 mmHg
Lactate	0.3 mmol/L	0.5-2.2 mmol/L
HCO3-	16.7 mmol/L	22-26 mmol/L
BE	-8.2 mmol/L	-2 to +2 mmol/L
Sodium	133 mmol/L	135-145 mmol/L
Potassium	4.23 mmol/L	3.5-5.0 mmol/L
Chloride	112 mmol/L	98-106 mmol/L

**Figure 3:**
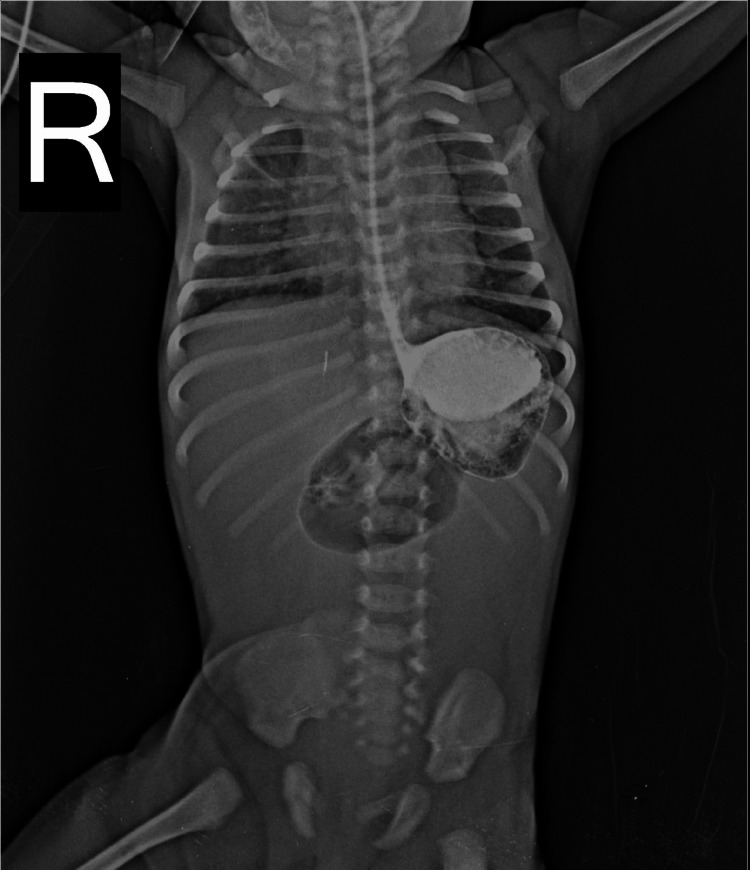
X-ray - erect abdomen (Case 2) A significant radiopaque structure in the right upper quadrant.

An X-ray of the erect abdomen was performed to further evaluate the abdominal distension and other presenting symptoms (Figure 3). The image revealed a significant radiopaque structure in the right upper quadrant. This finding is suggestive of a distended stomach or another organ that requires further investigation to determine the underlying cause of the observed abdominal distension.

A USG of the abdomen and pelvis revealed a distended stomach with both fluid and gas, along with a thickened muscular wall of the pylorus. The pylorus had a single muscle layer thickness of 4-5 mm, a pyloric length of 16-19 mm, a transverse diameter of 15 mm, and was minimally distensible. These findings were consistent with IHPS.

The initial management focused on the suspicion of late-onset sepsis, particularly due to the brown-colored RT aspirate and abdominal distension. Supportive care included the administration of IV fluids to stabilize the neonate and address her dehydration and feeding difficulties. The neonate's presentation, including symptoms such as abdominal distension and vomiting, along with laboratory findings of strongly positive C-reactive protein (CRP) and initial management steps, suggested a critical case of suspected late-onset sepsis. Notably, there were no significant metabolic disturbances detected. Given these observations, a thorough investigation and targeted therapeutic interventions were necessary. The treatment plan included a Ramstedt pyloromyotomy, which was performed uneventfully. Post-operatively, feeds were initiated 72 hours after surgery, marking the start of the recovery phase.

## Discussion

These cases highlight the clinical presentation and management of IHPS in the absence of the classic metabolic alkalosis typically associated with the condition. Instead of the usual hypochloremic, hypokalemic metabolic alkalosis, these infants exhibited either metabolic acidosis or no notable metabolic disturbances. This deviation from the expected electrolyte imbalance underscores the variability in both clinical and biochemical presentations of IHPS. Such observations emphasize the importance of maintaining a high index of suspicion and conducting thorough evaluations in infants presenting with symptoms like vomiting and dehydration, as these atypical presentations can still be indicative of IHPS.

Case 1: late preterm male neonate

The classic presentation of IHPS typically includes hypochloremic, hypokalemic metabolic alkalosis, as documented in literature due to the loss of gastric hydrochloric acid through persistent vomiting [8]. Hernanz-Schulman (2003) describes that IHPS usually affects infants between two and six weeks of age, presenting with non-bilious projectile vomiting and a palpable "olive-like" mass in the right upper quadrant [9]. The metabolic alkalosis observed in IHPS results from sustained vomiting, which leads to the loss of hydrochloric acid, sodium, and potassium, while bicarbonate reabsorption compensates for these losses.

In contrast, our first case involved a late preterm male neonate who presented with mild metabolic acidosis rather than the expected metabolic alkalosis. Despite the presence of projectile vomiting and a palpable abdominal mass, the metabolic acidosis suggests a more complex pathophysiology. This atypical presentation of metabolic acidosis in IHPS may indicate the presence of concurrent conditions, such as sepsis or severe dehydration, which could lead to poor perfusion and lactate accumulation [10]. Additionally, the initial USG report was misleading, showing normal findings, which underscores the importance of thorough and repeated diagnostic evaluation in the presence of atypical clinical presentations.

Case 2: term female neonate

The second case involved a term female neonate who was initially suspected of having late-onset sepsis due to her symptoms and the presence of brown-colored RT aspirate. Although IHPS usually presents with non-bilious vomiting, the brown-colored vomitus in this infant could be due to prolonged gastrointestinal stasis, which may lead to bleeding or bile-stained gastric contents from severe obstruction [11]. This atypical presentation underscores the variability in clinical manifestations of IHPS.

The infant’s blood gas analysis showed mild acidosis. The abdominal USG revealed a thickened pylorus and narrowed lumen, confirming the diagnosis of IHPS. The treatment plan included a Ramstedt pyloromyotomy, which is a standard and effective surgical intervention for IHPS [12]. Post-operatively, the infant was started on feeds 72 hours after surgery, following standard protocols for recovery.

The observation of metabolic acidosis in these cases deviates from the classical hypochloremic, hypokalemic metabolic alkalosis typically seen in IHPS [1]. This deviation can be attributed to several factors.

Prolonged vomiting can lead to significant dehydration and shock, potentially resulting in metabolic acidosis. The impaired tissue perfusion due to dehydration can cause anaerobic metabolism, which produces lactic acid and contributes to acidosis [4].

The presence of underlying infections or sepsis, as observed in the second case, can also result in metabolic acidosis. The initial symptoms mimicking late-onset sepsis underscore the importance of considering additional conditions that could influence the metabolic profile [13].

The biochemical profile of IHPS may vary based on the stage of the disease or atypical presentations. Early or non-classical cases of IHPS might not exhibit the traditional metabolic patterns, leading to differing biochemical findings, such as metabolic acidosis instead of the expected metabolic alkalosis.

These cases illustrate the need to consider IHPS in infants with significant vomiting and dehydration, even when metabolic alkalosis is not present. Clinicians should maintain a high level of suspicion and perform thorough evaluations, including imaging and surgical intervention, to ensure timely diagnosis and treatment.

## Conclusions

This case series of IHPS challenges the traditional view of the condition, which is typically associated with hypochloremic, hypokalemic metabolic alkalosis due to the loss of gastric acid from persistent vomiting. Instead, the cases presented here demonstrated metabolic acidosis, suggesting a broader spectrum of IHPS than previously recognized. This deviation may be attributed to severe dehydration, poor tissue perfusion, or concurrent conditions such as sepsis. The findings emphasize the importance of considering IHPS in infants with significant vomiting and dehydration, regardless of electrolyte imbalances. Early imaging, like abdominal USG, is crucial for diagnosis, and Ramstedt pyloromyotomy remains an effective treatment. This series underscores the need for flexible diagnostic approaches and comprehensive clinical evaluation to ensure timely and accurate management of IHPS.
